# Redox and Inflammatory Mechanisms in PMOS and Endometriosis: A Critical Review of Microbial and Plant‐Derived Interventions

**DOI:** 10.1002/iid3.70581

**Published:** 2026-11-11

**Authors:** Afnan Athar Hussain, Shafna Mohamed Ali, Ruaa Safwan Al Naser, Sahar Safari Abdullah, Mohamad Imadeddin Barakat, Sulagna Dutta, Pallav Sengupta, Motamarri Venkata Naga Lalitha Chaitanya, Prashanth Hegde, Shatabhisha Chakrabarti, Israel Maldonado Rosas, Shubhadeep Roychoudhury

**Affiliations:** ^1^ Department of Biomedical Sciences, College of Medicine Gulf Medical University Ajman UAE; ^2^ Basic Medical Sciences Department, College of Medicine Ajman University Ajman UAE; ^3^ Centre of Medical and Bio‐allied Health Sciences Research Ajman University Ajman UAE; ^4^ School of Pharmaceutical Sciences Lovely Professional University Jalandhar India; ^5^ Department of Obstetrics and Gynaecology Thumbay University Hospital Ajman UAE; ^6^ Health Centre Assam University Silchar India; ^7^ Department of Microbiology Green Heals Hospital Silchar India; ^8^ CITMER Reproductive Medicine Mexico City Mexico; ^9^ Department of Life Science and Bioinformatics Assam University Silchar India

**Keywords:** gynecological disorder, oxidative stress, phytochemicals, probiotics

## Abstract

**Background:**

Polycystic ovary syndrome (PCOS) or polyendocrine metabolic ovarian syndrome (PMOS) and endometriosis arise from different endocrine and tissue disturbances, yet oxidative stress (OS) and inflammation connect their initiating processes to reproductive dysfunction.

**Objective:**

In PMOS, redox‐inflammatory signaling intersects with insulin resistance, androgen excess, and follicular dysfunction; in endometriosis, it supports estrogen‐dependent lesion persistence, angiogenesis, and fibrosis. This narrative review examines these interactions and evaluates microbial and plant‐derived interventions according to their mechanistic targets and clinical evidence.

**Key Findings:**

Selected probiotic, synbiotic, and phytochemical trials in PMOS report improvements in metabolic, endocrine, and oxidative–inflammatory measures. In endometriosis, human intervention findings are concentrated in pain outcomes, while evidence for modulation of lesion biology remains predominantly experimental. Across both disorders, formulation, biological compartment, baseline phenotype, and concomitant treatment influence interpretation of therapeutic effects. Microbial metabolism offers a potential link between the two intervention classes by modifying phytochemical exposure and generating bioactive metabolites.

**Conclusions:**

The evidence‐informed comparative framework identifies where mechanistic findings have reached clinical evaluation and where further translation is needed. Future trials should connect standardized interventions and disease‐specific clinical outcomes with measurements of microbial function and redox signaling to establish which patients benefit and through which pathways.

## 1 Introduction

Endometriosis and polycystic ovary syndrome (PCOS), which has recently been renamed as polyendocrine metabolic ovarian syndrome (PMOS) together affect an alarming proportion of women of reproductive age, contributing substantially to global infertility and impaired quality of life [1]. Epidemiologically, PMOS impacts nearly 10%–15% of reproductive‐aged women worldwide [2], whereas endometriosis affects about 6%–10% [3]. Despite their distinct clinical manifestations, ectopic endometrial tissue growth in endometriosis and ovarian hyperandrogenism in PMOS, both disorders are associated with reproductive dysfunction, menstrual irregularity, metabolic derangements, and profound psychosocial consequences [4, 5]. Women often experience chronic pelvic pain, subfertility, depression, and anxiety, underscoring the complex interplay between physiological and emotional well‐being in these conditions [6, 7].

Recent decades have reframed both disorders as systemic diseases rather than organ‐confined pathologies. Mounting evidence identifies shared pathophysiological hallmarks: oxidative stress (OS), low‐grade chronic inflammation, and endocrine‐metabolic dysregulation [8, 9]. In endometriosis, excessive reactive oxygen species (ROS) from activated macrophages and iron overload promote inflammatory angiogenesis and tissue fibrosis [9]. Similarly, PCOS is marked by redox imbalance arising from hyperinsulinemia, adiposity, and mitochondrial dysfunction, perpetuating inflammatory cytokine release and androgen excess [10]. Reciprocal amplification between ROS generation and cytokine/NF‐κB (nuclear factor kappa‐light‐chain‐enhancer of activated B cells) activity provides a mechanistic intersection, while antioxidant responses shape the cellular consequences of that signaling. The different upstream drivers and tissue environments offer a useful comparison: the same molecular target may influence metabolic regulation in one disorder and lesion behavior in the other.

This review develops that comparison through microbial and plant‐derived interventions. Polyphenols, flavonoids, and carotenoids from dietary and medicinal plants, as well as antioxidant metabolites from gut microbiota, offer multitarget modulation of oxidative and inflammatory cascades [11]. Such bioactives not only restore redox equilibrium but also influence hormonal signaling, immune tone, and epigenetic regulators, dimensions highly relevant to the chronicity of endometriosis and PCOS [12, 13].

This narrative study synthesizes current evidence on the shared redox inflammatory mechanisms underlying PCOS and endometriosis and evaluates how microbial and plant‐derived antioxidants can target these convergent pathways. A structured literature search was conducted across major databases, including PubMed, Scopus, and Web of Science, focusing on studies addressing OS, inflammation, microbiome interactions, and antioxidant interventions in these disorders. Both experimental and clinical studies were critically appraised to integrate mechanistic insights with translational relevance. By relating intervention effects to disease context and outcome relevance, the review identifies the most informative directions for adjunctive treatment research.

## 2 OS and Inflammation as Common Results Denominators

### 2.1 Redox Biology in Female Reproductive Physiology

#### 2.1.1 ROS as Signaling Molecules in Folliculogenesis, Ovulation, and Implantation

ROS, traditionally regarded as harmful metabolic by‐products, also function as signaling mediators in female reproductive physiology [14]. Within the ovary, controlled ROS generation contributes to folliculogenesis, including follicular growth. Granulosa and theca cells generate ROS during follicular growth, modulating key intracellular pathways such as phosphoinositide 3‐kinase/protein kinase B (PI3K/Akt), mitogen‐activated protein kinase (MAPK), and NF‐κB that govern proliferation, differentiation, and steroidogenesis. These redox cues influence the balance between follicular survival and atresia, in coordination with hormonal control [15].

At ovulation, ROS act as messengers in the luteinizing hormone (LH) surge‐associated cascade [16]. Transient increases in ROS contribute to cumulus expansion, follicular rupture, and oocyte release through prostaglandin synthesis, matrix metalloproteinase activation, and vascular remodeling [17]. Experimental suppression of ROS during this periovulatory window can impair ovulation, highlighting their physiological signaling role [18]. When antioxidant buffering becomes insufficient, OS impairs oocyte quality and luteal function [14].

Beyond ovulation, ROS signaling extends to early embryo‐maternal crosstalk. Moderate ROS levels regulate endometrial receptivity through processes involving angiogenesis and extracellular matrix remodeling that support implantation [19]. Conversely, excessive or prolonged ROS exposure disrupts this delicate redox homeostasis, and can impair implantation and early embryonic development [14]. Reproductive function therefore depends on the timing, location, and magnitude of ROS production relative to antioxidant capacity. In conditions such as PMOS and endometriosis, chronic oxidative imbalance can disturb physiological redox signaling and promote cellular injury [10, 13]. This distinction provides a basis for evaluating microbial and plant‐derived redox modulators in relation to both oxidative injury and physiological reproductive signaling.

#### 2.1.2 Antioxidant Defense Systems

Reproductive tissues maintain redox homeostasis through coordinated enzymatic and non‐enzymatic defenses. Superoxide dismutase (SOD), catalase (CAT), glutathione peroxidase (GPx), and thioredoxin‐related systems act alongside glutathione (GSH) and antioxidant nutrients to regulate oxidant exposure across the ovarian and endometrial environments [20, 21, 22, 23, 24]. Their activity varies with tissue function and physiological state. Changes in antioxidant capacity in PMOS and endometriosis therefore require interpretation within the sampled compartment alongside measures of oxidative injury and reproductive function [13, 25, 26].

### 2.2 Dysregulated Redox Balance in PMOS

#### 2.2.1 Hyperandrogenism, Insulin Resistance, and Adiposity‐Related ROS

PMOS represents a complex endocrine‐metabolic disorder in which hyperandrogenism, insulin resistance (IR), and adiposity converge to create a self‐perpetuating redox‐inflammatory loop [27]. At the cellular level, these interlinked disturbances amplify ROS generation, disrupt mitochondrial homeostasis, and impair insulin signaling, culminating in systemic OS [10]. Hyperandrogenism, the clinical hallmark of PMOS, exerts profound effects on ovarian and peripheral redox balance. Excess androgens modulate nicotinamide adenine dinucleotide phosphate (NADPH) oxidase and mitochondrial electron transport chain (ETC) activity, enhancing superoxide production in granulosa and theca cells [28]. This aberrant ROS accumulation compromizes oocyte maturation, disrupts follicular vascularization, and triggers premature luteinization [29]. Simultaneously, hyperandrogenemia alters adipocyte differentiation, promoting visceral fat deposition and low‐grade inflammation that further fuels OS through cytokine‐driven ROS release [30].

IR, prevalent in nearly 70% of women with PMOS, compounds this oxidative burden. Impaired insulin signaling leads to reduced glucose uptake and enhanced lipolysis, elevating circulating free fatty acids that stimulate hepatic and adipose ROS production [31]. Chronic hyperinsulinemia, in turn, upregulates androgen synthesis via theca cell cytochrome P450c17 activity and suppresses sex hormone–binding globulin (SHBG), reinforcing the androgen excess [32].

Adiposity serves as both a source and a target of OS. Hypertrophic adipocytes and infiltrating macrophages generate ROS through NADPH oxidase activation and mitochondrial dysfunction, releasing pro‐inflammatory mediators such as tumour necrosis factor (TNF)‐α, interleukin (IL)−6, and C‐reactive protein (CRP) [33]. These cytokines exacerbate IR and inhibit antioxidant enzyme expression, diminishing systemic redox buffering capacity. The resulting oxidative‐inflammation cycle perpetuates follicular arrest, anovulation, and subfertility, hallmarks of PMOS pathophysiology [8].

#### 2.2.2 Altered Mitochondrial Function, Systemic Oxidative Load, and Circulating Biomarkers

Mitochondrial dysfunction is increasingly recognized as a central component of the redox and metabolic imbalance characteristic of PMOS [34]. As the primary site of oxidative phosphorylation, mitochondria regulate both energy metabolism and ROS generation; their impairment creates a vicious cycle of OS, metabolic inflexibility, and hormonal dysregulation [35]. In PCOS, structural and functional mitochondrial alterations, such as decreased membrane potential, fragmented cristae, and dysregulated fission–fusion dynamics, have been observed in oocytes, granulosa cells, and peripheral tissues, suggesting a systemic mitochondrial pathology rather than a localized ovarian defect [36].

Mitochondrial dysfunction, therefore, amplifies cellular oxidative damage and bioenergetic failure [10]. Mitochondrial DNA (mtDNA), being particularly vulnerable due to its proximity to the ETC and lack of protective histones, accumulates mutations that further exacerbate bioenergetic inefficiency [37]. Reduced ATP synthesis impairs oocyte maturation, spindle integrity, and embryonic competence, key determinants of reproductive failure in PMOS [36]. Meanwhile, systemic oxidative overload extends beyond the ovary, affecting skeletal muscle, adipose tissue, and endothelium, where redox imbalance impairs insulin signaling and vascular function [25]. Several circulating biomarkers mirror this heightened oxidative burden. Multiple OS biomarkers reflect systemic redox imbalance in PMOS [38]. Simultaneously, antioxidant defenses, including SOD, CAT, and GPx, are often reduced or functionally compromized. Increased total oxidant status (TOS) and decreased total antioxidant capacity (TAC) collectively define the OS index, a useful systemic marker of redox imbalance in PMOS [39]. This mitochondrial‐redox interplay underscores PMOS as a multisystem disorder of bioenergetic dysfunction. Targeting mitochondrial health through microbial‐ and plant‐derived antioxidants, such as polyphenols, coenzyme Q10 (CoQ10), and microbial short‐chain fatty acids (SCFAs), offer promising therapeutic potential to restore redox homeostasis, enhance insulin sensitivity, and improve reproductive outcomes in affected women.

### 2.3 Dysregulated Redox Balance in Endometriosis

Endometriosis represents a chronic, estrogen‐dependent inflammatory disorder in which dysregulated redox homeostasis serves as both an initiating and perpetuating factor in disease progression. The ectopic implantation of endometrial cells into the peritoneal cavity exposes them to an oxidative microenvironment rich in heme, free iron, and activated immune cells. Retrograde menstruation introduces erythrocytes and haemoglobin into the peritoneal cavity, where their breakdown releases free iron that drives Fenton reactions and activates macrophages, leading to excessive ROS generation and the establishment of a persistent oxidative and inflammatory microenvironment that supports lesion survival and progression.

In physiological conditions, ROS play signaling roles in cyclic endometrial remodeling, angiogenesis, and implantation. However, in endometriosis, this transient signaling becomes chronic and destructive. The excessive ROS burden overwhelms antioxidant defenses. Reduced expression or activity of these antioxidants has been observed in the eutopic and ectopic endometria of affected women. The imbalance between ROS generation and detoxification promotes lipid peroxidation (LPO), protein carbonylation, and oxidative DNA damage, hallmarks of cellular stress that foster genomic instability and aberrant cell proliferation.

Mitochondrial dysfunction is another defining feature of redox dysregulation in endometriosis. Altered mitochondrial membrane potential and impaired oxidative phosphorylation compromise adenosine triphosphate (ATP) synthesis while amplifying electron leakage, thereby increasing mitochondrial ROS output. Damaged mitochondria, in turn, release mtDNA into the cytosol and peritoneal fluid, triggering innate immune activation via pattern recognition receptors and reinforcing inflammation. This oxidative‐inflammatory feedback loop facilitates the survival and invasiveness of ectopic endometrial cells while impairing apoptotic clearance.

At the molecular level, ROS activate key transcriptional regulators such as NF‐κB and hypoxia‐inducible factor‐1α (HIF‐1α), driving the expression of pro‐inflammatory cytokines (IL‐1β, IL‐6, TNF‐α), adhesion molecules, and angiogenic mediators like vascular endothelial growth factor (VEGF). The resulting microenvironment favours neoangiogenesis, fibrogenesis, and immune evasion, central events in lesion persistence. Furthermore, estrogen itself reinforces OS through redox cycling of its catechol metabolites, sustaining a vicious cycle between hormonal excess and oxidative injury. The cumulative effect of these processes is a self‐perpetuating redox‐inflammatory circuit that underlies the chronicity and recurrence of endometriosis. Understanding these redox perturbations provides a mechanistic rationale for antioxidant‐based interventions, particularly those utilizing microbial‐ and plant‐derived bioactives, to restore mitochondrial function, suppress oxidative signaling, and re‐establish redox equilibrium within the peritoneal niche.

### 2.4 Inflammation‐Redox Crosstalk in Both Disorders

#### 2.4.1 Cytokine Networks (IL‐6, TNF‐α, IL‐1β)

Inflammatory cytokines connect immune activation with OS and endocrine dysfunction in PMOS and endometriosis [8, 40]. Among these mediators, IL‐6, TNF‐α, and IL‐1β participate in feedback loops in which inflammatory signaling increases ROS production and OS further modifies cytokine responses. Their effects depend on cellular source and tissue environment, linking metabolic inflammation to ovarian dysfunction in PMOS and local inflammatory activity to lesion persistence in endometriosis.

In PMOS, adipose tissue inflammation and macrophage infiltration contribute to IL‐6 and TNF‐α production. TNF‐α impairs insulin signaling through mechanisms involving inhibitory serine phosphorylation of insulin receptor substrate‐1 (IRS‐1), thereby aggravating IR [41]. IL‐6 contributes to hepatic CRP production and has been associated with changes in SHBG, connecting inflammatory activity with androgen bioavailability [42]. IL‐1β also participates in local ovarian inflammatory responses, including leukocyte recruitment and processes affecting granulosa‐cell survival and follicular development [43].

In endometriosis, activated macrophages and ectopic endometrial stromal cells contribute to a peritoneal cytokine environment enriched in IL‐6, TNF‐α, and IL‐1β [44]. These mediators influence immune‐cell recruitment, stromal‐cell behaviour, and tissue remodeling. IL‐1β promotes cyclooxygenase‐2 (COX‐2) expression and prostaglandin E_2_ production, connecting cytokine activity with pain and local inflammation [45]. Cytokine‐driven ROS production further reinforces inflammatory injury and remodeling within the lesion microenvironment [46].

The relative contributions of these cytokines vary across anatomical compartments, with metabolic inflammation prominent in PMOS and lesion‐associated inflammation prominent in endometriosis [47].

#### 2.4.2 NF‐κβ and Nrf2 Pathways

NF‐κB and nuclear factor erythroid 2‐related factor 2 (Nrf2) connect inflammatory transcription with cellular antioxidant responses [48]. NF‐κB regulates genes involved in inflammation, cell survival, and tissue remodeling, whereas Nrf2 coordinates antioxidant and cytoprotective programmes. Their interactions shape cellular responses to oxidative and inflammatory stimuli.

In the canonical NF‐κB pathway, inhibitor of κB (IκB) proteins retain NF‐κB in the cytoplasm. Activation of upstream signaling promotes IκB phosphorylation and degradation, permitting NF‐κB nuclear translocation and transcription of mediators such as TNF‐α, IL‐6, COX‐2, and inducible nitric oxide synthase (iNOS) [49]. In PMOS, metabolic stimuli associated with hyperinsulinemia and elevated free fatty acids can engage NF‐κB signaling in ovarian and adipose tissues, connecting metabolic stress with inflammatory transcription [50]. In endometriosis, NF‐κB activity in ectopic stromal cells and immune cells contributes to inflammatory, adhesive, and angiogenic processes that support lesion persistence [51, 52].

Nrf2 regulates antioxidant response element (ARE)‐dependent transcription, including expression of heme oxygenase‐1 (HO‐1), NAD(P)H quinone oxidoreductase 1 (NQO1), and enzymes involved in glutathione synthesis and metabolism [53]. The effectiveness of this response depends on cell type, disease stage, and the intensity and duration of oxidative exposure. Altered Nrf2 signaling has been implicated in PMOS and endometriosis, linking antioxidant adaptation with mitochondrial stress and inflammatory activity [54, 55].

Experimental studies indicate that selected phytochemicals and microbial metabolites can influence both pathways. Curcumin, resveratrol, sulforaphane, and SCFAs have been investigated for effects on NF‐κB‐dependent inflammation and Nrf2‐associated antioxidant responses [56, 57, 58, 59]. These findings provide mechanistic targets for the intervention studies discussed in Sections 3 and 4.

#### 2.4.3 Endocrine‐Immune‐Metabolic Convergence

In PMOS, hyperandrogenism, IR, and inflammation interact through reciprocal endocrine–metabolic feedback (Table 1). Hyperinsulinaemia promotes ovarian theca‐cell androgen synthesis through steroidogenic pathways involving cytochrome P450c17 (CYP17A1) and augments LH responsiveness [8, 60]. Excess androgens impair follicular development and favour visceral adiposity, while reduced adiponectin signaling further connects endocrine disturbance with metabolic dysfunction [8, 61]. These interactions link peripheral IR to ovarian steroidogenesis and reproductive function.

**Table 1:** Shared redox‐inflammatory pathways linking PCOS (or PMOS) and endometriosis and potential nutraceutical targets.

Pathophysiological axis	Key Molecular Mediators	Role in PCOS/PMOS	Role in endometriosis	Potential microbial/Plant interventions	References
Oxidative stress	ROS, malondialdehyde (MDA), mitochondrial dysfunction	Impaired folliculogenesis, IR	Lesion survival, peritoneal damage	Polyphenols, carotenoids, probiotics	Agarwal et al., 2012
Inflammatory signaling	NF‐κβ, TNF‐α, IL‐6	Chronic low‐grade inflammation	Peritoneal inflammatory milieu	Curcumin, resveratrol, SCFAs	Vallée et al., 2021
Hormonal dysregulation	Insulin, androgens, estrogen	Hyperandrogenism, IR	Estrogen‐driven lesion growth	Probiotics, resveratrol, quercetin	Banaszewska et al., 2016
Angiogenesis	VEGF, COX‐2	Ovarian stromal remodeling	Lesion vascularization	Epigallocatechin‐3‐gallate (EGCG), curcumin	Xu et al., 2013
Gut‐reproductive axis	Lipopolysaccharide (LPS), SCFAs, microbial metabolites	Dysbiosis‐driven metabolic inflammation	Microbiome–immune dysregulation	Lactobacillus/Bifidobacterium probiotics	Guo et al., 2024

In endometriosis, local estrogen signaling and progesterone resistance intersect with immune dysregulation to sustain ectopic endometrial‐like tissue [62, 63]. Estrogen‐responsive pathways influence macrophage phenotype and interact with inflammatory and angiogenic signaling within the lesion microenvironment [64]. Prostaglandin E_2_ stimulates aromatase‐dependent local estrogen biosynthesis, creating feedback between inflammatory activity and estrogen production [63]. OS and NF‐κB‐dependent signaling further contribute to the inflammatory environment supporting lesion persistence [64, 65].

Endocrine signaling and inflammation therefore reinforce one another in both disorders, but through different dominant circuits: an insulin–androgen interaction in PMOS and an estrogen–inflammation interaction in endometriosis [61, 63]. These circuits provide a comparative framework for understanding how overlapping downstream pathways produce distinct metabolic and tissue consequences (Figure 1).

**Figure 1:**
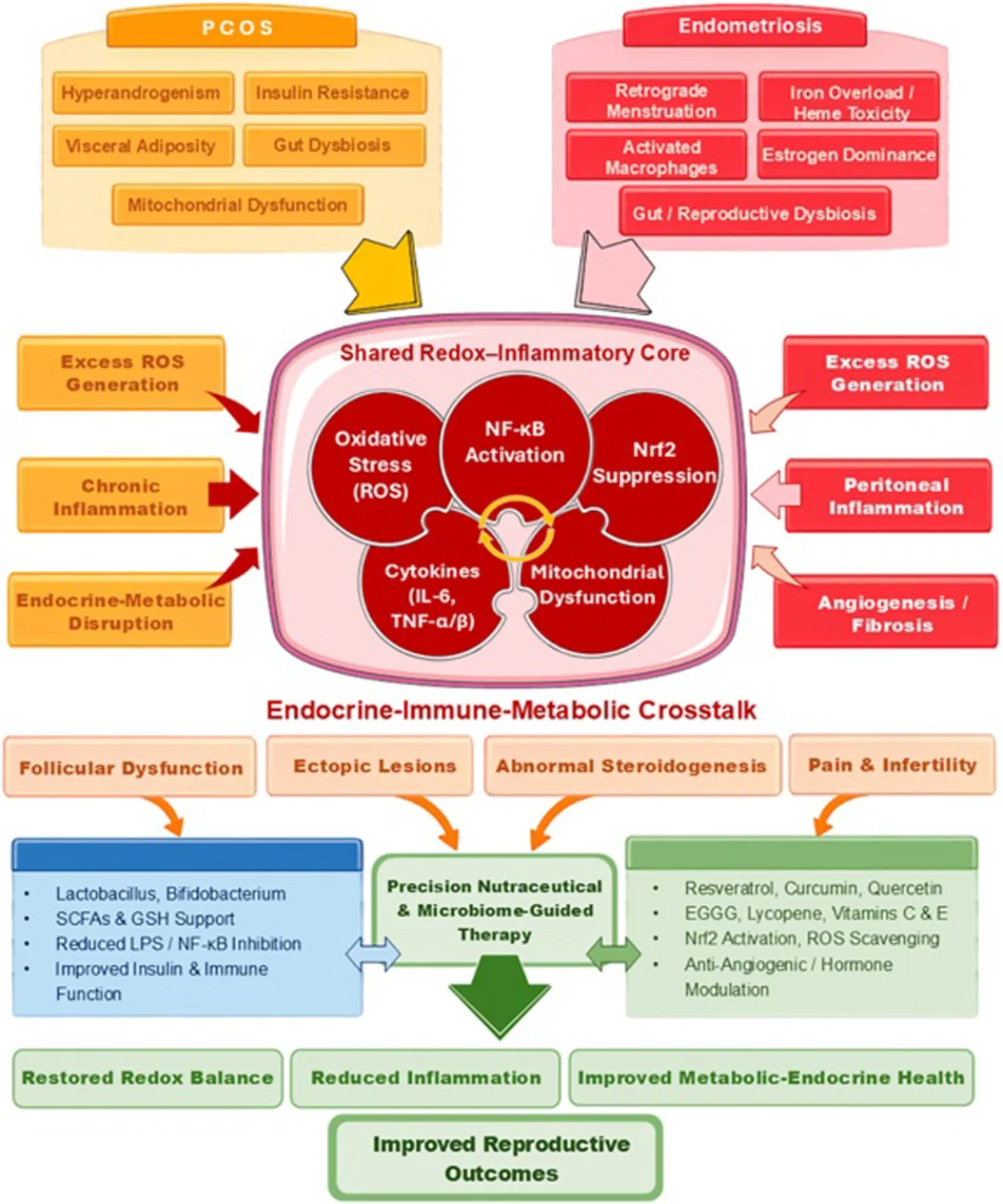
Integrated schematic of the redox–inflammatory convergence linking PCOS/PMOS and endometriosis, and the modulatory roles of microbial‐ and plant‐derived antioxidants. Disorder‐specific upstream triggers in PCOS/PMOS and endometriosis converge on a shared oxidative‐inflammatory core characterized by ROS excess, cytokine activation, NF‐κB/Nrf2 imbalance, mitochondrial dysfunction, and endocrine‐immune‐metabolic crosstalk. Microbial‐derived antioxidants and plant‐derived phytochemicals may intercept these pathways through antioxidant, anti‐inflammatory, barrier‐protective, anti‐angiogenic, and hormone‐modulating mechanisms, supporting precision adjunctive therapy in reproductive medicine.

## 3 Microbial‐Derived Antioxidants in PMOS and Endometriosis

### 3.1 Gut‐Reproductive Axis: Microbiome Dysbiosis in PMOS and Endometriosis

The gut microbiome comprises a highly complex community of microorganisms inhabiting the gastrointestinal tract and contributes fundamentally to host homeostasis through effects on nutrient metabolism, epithelial integrity, immune regulation, and endocrine signaling [66, 67]. Within this broader framework, the gut‐reproductive axis refers to the bidirectional communication between intestinal microbial communities and the female reproductive system through interconnected immune, metabolic, neuroendocrine, and hormonal pathways [66, 68]. Dysbiosis, broadly defined as a disruption in the composition, diversity, or function of the microbiome, has been linked to several reproductive disorders, including PMOS and endometriosis, plausibly through immune dysregulation, systemic inflammation, altered steroid metabolism, and disturbed hypothalamic‐pituitary‐gonadal (HPG) signaling [67, 69].

The gut‐reproductive axis has received growing attention in PMOS, a disorder characterized by hyperandrogenism, IR, ovulatory dysfunction, and chronic low‐grade inflammation. Human studies and systematic reviews indicate that women with PMOS exhibit consistent compositional alterations in the gut microbiota, although findings on alpha‐diversity are not fully uniform across studies [70, 71]. Rather than claiming a universal reduction in diversity, current evidence more safely supports the presence of gut microbial restructuring, with altered abundance of taxa linked to inflammatory tone, androgen status, and metabolic dysfunction [70, 71]. These dysbiotic patterns are thought to contribute to the persistence of IR, hyperandrogenism, and metabolic syndrome, partly through lipopolysaccharide (LPS)‐mediated inflammation, altered SCFA signaling, bile acid perturbation, and interactions with sex hormone metabolism [72, 73].

Endometriosis has likewise been associated with microbiome disturbance, although the evidence base is somewhat more heterogeneous and spans both the gut and the reproductive tract microbiota. Available reviews support an association between endometriosis and dysbiosis, with plausible links to immune dysfunction, inflammatory activation, and altered estrogen handling [74, 75, 76]. Importantly, reports of reduced Lactobacillus and enrichment of taxa such as Gardnerella are more consistently described in the vaginal/cervicovaginal or endometrial microbiota, rather than being established as universal gut signatures of endometriosis [77, 78]. Thus, in endometriosis, it is more accurate to state that dysbiosis across the gut‐reproductive microbial axis may contribute to disease progression, rather than attributing all such taxa‐specific findings specifically to the gut compartment.

Human microbiome findings in endometriosis primarily establish associations, with temporal relationships influenced by diet, medication, gastrointestinal symptoms, and sampling compartment. Barrier‐associated immune signaling and microbial estrogen deconjugation provide candidate mechanisms linking intestinal microbial activity with disease biology [67, 75, 76, 79, 80] establishing their contribution to lesion progression requires longitudinal and intervention studies that connect microbial function with clinical outcomes.

### 3.2 Probiotic Strains and Microbial Products in Redox Regulation

#### 3.2.1 *Lactobacillus* and *Bifidobacterium* strains‐producing SCFAs, glutathione, and exopolysaccharides (EPS)

Selected strains of *Lactobacillus* and *Bifidobacterium* have metabolic, immunomodulatory, and antioxidant properties relevant to the investigation of PMOS and endometriosis. Their fermentation activities can influence intestinal organic‐acid profiles directly and through interactions with other microorganisms. These contributions depend on strain characteristics, available substrates, and microbial community composition. In PMOS, altered microbial metabolite profiles, including SCFA‐related pathways, have been associated with IR, androgen excess, and inflammatory activity [66, 81].

Beyond fermentation, selected Lactobacilli and Bifidobacteria possess antioxidant‐associated systems involving thiol metabolism, GSH‐related machinery, and bacterial redox enzymes. Their metabolites and structural components have also shown ROS‐scavenging, metal‐chelating, and host antioxidant‐modulating activity in experimental systems [82, 83]. These properties vary among strains and distinguish microbial antioxidant activity from changes in host antioxidant defenses.

EPS provide a further source of microbial bioactivity. EPS derived from *Lactobacillus rhamnosus* GG have demonstrated radical‐scavenging and ferrous‐ion‐chelating activity, protection against oxidative injury, and modulation of Kelch‐like ECH‐associated protein 1 (Keap1)/Nrf2 signaling in intestinal epithelial models [84]. Such findings identify EPS as candidate mediators of epithelial redox regulation, with their relevance to ovarian and endometriotic tissue requiring disease‐specific investigation.

#### 3.2.2 Anti‐Inflammatory and ROS‐Scavenging Potential

Microbial products influence host redox status through both direct chemical activity and regulation of epithelial and immune responses. SCFAs act through receptor‐mediated and epigenetic mechanisms, including G‐protein‐coupled receptor (GPCR) signaling and histone deacetylase inhibition. Butyrate, in particular, has demonstrated effects on inflammatory signaling and epithelial barrier function in experimental systems [85]. Its immunomodulatory activity includes the induction of IL‐10‐producing plasma cells in murine experiments [86]. Improved barrier integrity can limit exposure to microbial inflammatory products, connecting intestinal microbial activity with systemic immune and metabolic responses [85].

GSH‐associated pathways contribute to cellular redox buffering, while changes in host antioxidant systems can modify susceptibility to oxidative injury [82, 83]. Clinical increases in GSH or TAC and reductions in malondialdehyde (MDA) reflect host biochemical responses; identifying the microbial contribution that requires concurrent measurements of strain activity and metabolite production. In women with PMOS, probiotic supplementation increased TAC and reduced MDA, while probiotic–selenium co‐supplementation increased both TAC and GSH and reduced MDA [87, 88]. The latter findings concern the combined preparation and do not isolate the probiotic contribution.

EPS‐associated effects include radical scavenging, metal chelation, and modulation of cytoprotective signaling. EPS from *L. rhamnosus* GG demonstrated antioxidant activity and protection against oxidative injury in intestinal epithelial models [84]. EPS from *L. delbrueckii* subsp. *bulgaricus* 1.0207 also attenuated hydrogen peroxide‐induced injury in IPEC‐J2 (intestinal porcine enterocyte cell line ‐ jejunum 2) cells through effects involving the Keap1/Nrf2 pathway [89]. Together with SCFA‐mediated immune regulation, these activities identify complementary routes through which microbial products may influence oxidative and inflammatory processes. Their contribution to therapeutic effects in PMOS and endometriosis can be investigated by measuring microbial metabolites, barrier‐associated markers, and redox responses alongside disease‐specific clinical outcomes.

### 3.3 Evidence from Experimental Models and Clinical Trials

#### 3.3.1 Probiotic Interventions in PMOS: Insulin Sensitivity, Hormonal Modulation, and OS Markers

Experimental studies provide evidence linking microbial interventions with metabolic and reproductive function in PMOS models. In a letrozole‐induced rat model, *Lacticaseibacillus paracasei* subsp. *paracasei* DSM 27449 improved ovarian function, reduced cystic follicles, and lowered serum testosterone [90]. In a dihydrotestosterone (DHT)‐induced rat model, probiotic treatment improved reproductive and metabolic abnormalities alongside changes in gut microbial composition [91]. Complementary evidence comes from microbial‐exposure experiments. Cohousing letrozole‐treated mice with placebo‐treated mice protected against the development of metabolic and reproductive abnormalities [92], whereas transplantation of foecal microbiota from women with PCOS into germ‐free mice induced metabolic disturbances and ovarian dysfunction [93]. These experiments support a functional contribution of the microbiota within the models studied and provide a basis for evaluating microbial interventions in women with PCOS.

Human randomized trials, commonly lasting 8–12 weeks, have examined insulin‐related outcomes using defined probiotic or synbiotic preparations. Evidence syntheses report reductions in fasting glucose, fasting insulin, and HOMA‐IR (Homeostasis Model Assessment‐estimated Insulin Resistance), although the magnitude and consistency of these effects vary across interventions and study populations [94, 95]. Probiotic and synbiotic preparations have each produced favourable findings for selected glycaemic measures, but differences between studies do not establish comparative superiority [94, 96]. Formulation and baseline metabolic characteristics remain important considerations when interpreting these responses.

Hormonal findings are similarly outcome‐specific. A 12‐week placebo‐controlled probiotic trial reported increased SHBG and reduced total testosterone and modified Ferriman–Gallwey scores [87]. A separate 12‐week synbiotic trial reported increased SHBG and reduced modified Ferriman–Gallwey scores, without significant changes in the other measured hormonal outcomes [97]. Evidence syntheses also identify changes in selected androgen‐related measures, with variation across preparations and studies [95, 98]. Strain composition, dose, treatment duration, adiposity, dietary context, and concomitant treatment may contribute to this variation. Biochemical androgen changes and short‐term hirsutism‐score changes should be evaluated separately from sustained improvements in ovulatory function.

Oxidative and inflammatory outcomes provide an additional level of evidence. The probiotic trial reported increased TAC and reduced MDA and high sensitivity (hs)‐CRP [87]. The synbiotic trial reported increased nitric oxide (NO) and reduced hs‐CRP, without significant changes in TAC, GSH, or MDA [97]. In a further 12‐week trial, probiotic–selenium co‐supplementation increased TAC and GSH and reduced MDA and hs‐CRP [88]. These findings concern the combined preparation and do not isolate the probiotic contribution. Collectively, the studies demonstrate preparation‐specific changes in host biochemical measures; whether these changes mediate metabolic or reproductive improvement requires concurrent assessment of microbial function, metabolite exposure, and clinical outcomes.

Evidence syntheses also report benefits for selected anthropometric and lipid outcomes, although findings differ by intervention and outcome, and certainty is constrained by the size, duration, and methodological quality of the underlying studies [94, 96]. The available findings support further evaluation of defined probiotic and synbiotic preparations as adjunctive interventions, with attention to insulin‐related and androgen‐related outcomes [95]. Future trials should characterize baseline IR and androgen status alongside adiposity, standardize preparations and doses, and assess ovulatory function and other relevant clinical outcomes. Integrated microbial, metabolite, and redox measurements would help identify response mechanisms, while follow‐up beyond the usual 8–12‐week treatment period would establish the durability of benefit.

#### 3.3.2 Probiotic/Prebiotic/Postbiotic Interventions in Endometriosis: Immune Regulation, Pain, and OS Biomarkers

Experimental studies have examined microbial interventions in relation to lesion development and immune responses. In a murine endometriosis model, oral administration of heat‐killed *L. gasseri* OLL2809 suppressed ectopic lesion development, with findings implicating IL‐12‐associated stimulation of natural killer (NK) cell activity [99]. This preparation provides an example of bacterial‐product‐mediated immune modulation, distinct from interventions intended to alter intestinal colonization.

Human intervention findings have focused principally on pain. A randomized, double‐blind, placebo‐controlled trial of heat‐killed *L. gasseri* OLL2809 administered for 12 weeks reported improvements in menstrual pain intensity and dysmenorrhea [100]. A subsequent pilot placebo‐controlled trial evaluated a live multistrain Lactobacillus preparation over 8 weeks and reported reductions in dysmenorrhea and an aggregate pain measure, while between‐group differences in dyspareunia and chronic pelvic pain were less consistent [101]. These studies identify preparation‐specific symptom effects; they do not establish corresponding changes in lesion burden or the biological mechanisms underlying pain improvement.

Microbial metabolite research provides a separate line of experimental evidence. Administration of n‐butyrate reduced lesion growth in mouse models and inhibited survival of human endometriotic epithelial cells in vitro [102]. Mechanistic experiments implicated GPCR 43 (GPR43) and GPCR 109 A (GPR109A) signaling, histone deacetylase inhibition, and increased expression of RAP1GAP [Ras‐like guanosine triphosphate (GTP)ase 1 GTPase‐activating protein], a negative regulator of pro‐growth RAP1 (Ras‐like GTPase 1) signaling. Partial reversal of the response following receptor blockade indicated contributions from additional pathways. These findings establish an experimental effect on lesion‐associated growth processes and identify butyrate‐responsive signaling as a target for further investigation.

The relationship between these intervention effects and redox regulation remains insufficiently characterized. Observational studies have reported increased oxidative damage measures in serum, peritoneal fluid, and ovarian tissue, although findings depend on the marker and compartment examined [103, 104, 105]. The clinical bacterial‐preparation studies described above did not establish whether pain improvement was accompanied by changes in these measures [100, 101]. Pairing symptom assessment with compartment‐appropriate oxidative and inflammatory markers would help determine whether redox modulation contributes to the observed clinical response.

Direct clinical evaluation of prebiotic interventions in endometriosis remains limited [80, 109]. Their contribution should be investigated through measured changes in microbial function and clinical outcomes rather than inferred from fermentation biology alone. The ProMetrioS (The Role of Probiotics in the Treatment of Endometriosis) protocol extends this approach by evaluating a defined multispecies probiotic preparation alongside gut microbial composition and function, including estrobolome‐related parameters [106]. As a protocol, it specifies an investigational strategy rather than evidence of treatment efficacy. Linking pain and quality of life outcomes with microbial functional measures would help distinguish symptom effects from changes in the proposed gut–endometriosis pathways.

### 3.4 Challenges and Opportunities

The therapeutic promise of microbial‐derived antioxidants, principally probiotics, postbiotics, and metabolite‐centered microbiome interventions, has gained traction in both PMOS and endometriosis because these strategies can influence inflammatory signaling, epithelial barrier integrity, estrogen handling, and redox homeostasis through pathways linked to SCFAs, antioxidant enzymes, and other bioactive microbial products [66, 75, 79, 107] (Table 2). In PMOS, clinical and preclinical studies suggest that microbiota‐targeted interventions may improve IR, inflammatory tone, and selected OS markers, whereas in endometriosis the most compelling signals currently relate to pain modulation, SCFA‐mediated anti‐lesion mechanisms, and broader immune‐endocrine regulation [75, 95, 102, 108]. However, translation into routine care remains constrained by major challenges involving strain specificity, dose selection, delivery technology, host heterogeneity, and standardization of trial endpoints [95, 109].

**Table 2:** Microbial and plant‐derived antioxidants targeting the redox–inflammatory axis in PCOS and endometriosis.

Intervention	Key bioactive components	Major mechanisms	Evidence mModel	Key outcomes reported	Key references
Probiotics (*Lactobacillus, Bifidobacterium*)	SCFAs, EPS, glutathione	Gut barrier restoration; SCFA‐mediated GPCR signaling; immune modulation; reduced LPS inflammation	Animal models, randomized controlled trials (RCTs)	Improved insulin sensitivity, reduced inflammatory markers, improved hormonal balance	He et al., 2023; Guo et al., 2024
Resveratrol	Stilbene polyphenol	NF‐κB inhibition; Nrf2 activation; anti‐angiogenic; steroidogenesis suppression	Clinical and experimental	Reduced testosterone, improved insulin sensitivity, reduced lesion inflammation	Banaszewska et al., 2016; Ricci et al., 2013
Curcumin	Curcuminoids	ROS scavenging; NF‐κB suppression; anti‐angiogenic; cytokine inhibition	Animal models, small clinical studies	Improved metabolic parameters; reduced inflammatory signaling	Panahi et al., 2017; Vallée et al., 2021
Catechins (EGCG)	Green tea polyphenols	Anti‐angiogenic (VEGF inhibition); antioxidant enzyme activation	Animal studies	Reduced endometriotic lesion growth; improved metabolic markers	Xu et al., 2013
Quercetin	Flavonoid	Anti‐inflammatory signaling; apoptosis induction; insulin signaling modulation	Experimental and animal studies	Reduced androgen production; inhibited ectopic cell proliferation	Wang et al., 2017
Carotenoids/Vitamins (lycopene, vitamin E)	Lipid‐phase antioxidants	LPO inhibition; membrane protection; improved TAC	Clinical and animal studies	Reduced MDA; improved oxidative biomarkers	Asemi et al., 2015; Mier‐Cabrera et al., 2009

A central limitation is, however, strain specificity. Probiotic effects are not class effects; they are strain‐dependent and shaped by the host ecological context. Reviews of probiotic biology consistently emphasize that different strains vary in their ability to modulate NF‐κB, reinforce epithelial barrier function, influence oxidative pathways, or interact with host

immunity and metabolism, making it scientifically unsafe to extrapolate findings from one strain to another, even within the same species [82, 108, 109]. This is especially relevant in reproductive disorders, where the endocrine milieu, baseline dysbiosis pattern, adiposity, diet, and background microbiota may all influence colonization resistance, metabolite output, and therapeutic response [66, 75]. Accordingly, a strain that appears useful in metabolically driven PMOS may not perform similarly in endometriosis, where estrogen dependence, immune escape, and lesion biology dominate the phenotype [66, 107].

A second challenge is dose, formulation, and delivery. Oral probiotics often face substantial attrition during passage through gastric acid, bile salts, oxygen exposure, storage, and food‐processing conditions, all of which can reduce viability and downstream efficacy [110, 111]. Newer approaches such as microencapsulation, synbiotic pairing with fermentable fibres, and postbiotic delivery seek to improve microbial survival, targeted release, and functional consistency, but the clinical evidence supporting these strategies in gynaecologic disorders remains early rather than definitive [110, 112]. This means that dose‐response relationships, therapeutic windows, and formulation‐dependent bioavailability still need to be established before these approaches can be rationally standardized for PMOS or endometriosis care [95, 110].

A related opportunity lies in moving from live probiotics alone toward next generation synbiotic and postbiotic strategies. SCFAs such as butyrate are particularly attractive because they can influence GPCR signaling, histone deacetylase activity, epithelial integrity, inflammatory cytokine output, and oxidative pathways, thereby acting at the interface of metabolism, immunity, and redox regulation [102, 107, 113]. In PMOS, SCFA‐centered approaches may intersect with insulin sensitivity and low‐grade inflammation; in endometriosis, they may influence lesion proliferation, immune surveillance, and inflammatory microenvironment signaling [102, 108, 113]. This creates a strong rationale for designing interventions that do not merely add live microbes, but instead deliberately shape metabolite flux through fibre‐supported fermentation, defined consortia, or direct postbiotic supplementation [112, 113].

Integration with standard therapy presents both opportunity and complexity. Probiotics or postbiotics may eventually complement insulin sensitizers, hormonal therapies, or anti‐inflammatory drugs by reducing inflammatory burden and improving metabolic resilience, but this cannot be assumed to be uniformly beneficial or interaction‐free [95, 114]. The gut microbiome can alter drug metabolism, and drugs can reshape microbiome composition, raising the possibility of clinically relevant pharmacomicrobiomic interactions that remain largely unexplored in PMOS and endometriosis [114, 115]. For that reason, future translational programmes should not treat microbial therapeutics as isolated wellness adjuncts; they should be studied as biologically active co‐interventions with possible effects on pharmacokinetics, efficacy, tolerability, and host‐response variability [112, 114].

The most important opportunity is precision microbiome therapeutics. Robust multi‐omics profiling, including metagenomics, metabolomics, redox biomarker panels, and endocrine‐immune phenotyping, could help identify which patients are most likely to benefit and which microbial functions should be targeted [95, 112]. For PMOS, this may mean stratifying by obesity, IR, androgen excess, and baseline SCFA‐related dysbiosis; for endometriosis, it may require integrating lesion phenotype, pain pattern, inflammatory signatures, and estrogen‐metabolic context [75, 107]. In that sense, the long‐term potential of the field lies not in generic probiotic prescribing, but in developing formulation‐specific, phenotype‐matched interventions tailored to the redox‐inflammatory architecture of each disorder [109, 112]. At present, therefore, microbial therapeutics are best viewed as promising adjunctive tools with real mechanistic credibility, but not yet as standardized core therapy.

## 4 Plant‐Derived Antioxidants in PMOS and Endometriosis

### 4.1 Polyphenols and Flavonoids

#### 4.1.1 Resveratrol, Quercetin, Catechins, and Curcumin

PCOS is primarily defined by hyperandrogenism, ovulatory dysfunction, and IR, whereas endometriosis is an estrogen‐dependent inflammatory disease marked by ectopic endometrial‐like tissue growth, pelvic pain, and infertility. In this context, plant‐derived polyphenols such as resveratrol, quercetin, catechins, and curcumin have attracted attention for their multitarget bioactivity [66, 116, 117].

Resveratrol, a stilbene abundant in grapes and red wine, is among the best studied polyphenols in PMOS. In a double‐blind randomized placebo‐controlled trial, resveratrol significantly reduced total testosterone and dehydroepiandrosterone sulfate (DHEAS) concentrations and improved fasting insulin‐related measures in women with PMOS, supporting an effect on both androgen excess and insulin sensitivity [118]. Mechanistically, experimental studies indicate that resveratrol can suppress ovarian androgen biosynthesis by inhibiting CYP17A1 expression or activity in theca‐interstitial cells, thereby reducing steroidogenesis rather than merely masking endocrine symptoms [119, 120]. In endometriosis, resveratrol has shown anti‐inflammatory and anti‐angiogenic actions in experimental systems; animal and cell‐based studies indicate inhibition of angiogenesis, inflammatory mediators, and lesion‐supportive pathways, although clinical confirmation remains limited [116, 121, 122].

Quercetin, a flavonol found in apples, onions, and many fruits and vegetables, also shows multi‐target potential in PMOS. Human supplementation studies have reported improvements in adiponectin‐mediated IR pathways and reductions in testosterone and LH levels in overweight or obese women with PMOS, while animal studies support additional benefits on ovarian morphology, folliculogenesis, and metabolic function [123, 124, 125, 126]. Thus, the most defensible conclusion is that quercetin may improve both metabolic and endocrine features of PMOS, although the magnitude of benefit remains context dependent. In endometriosis, quercetin has demonstrated antiproliferative activity in preclinical models. It inhibits proliferation, induces cell‐cycle arrest, and promotes apoptosis, with reported DNA fragmentation, loss of mitochondrial membrane potential, reduced CCND1 (cyclin D1) expression, and altered microRNA (miRNA) signaling in endometriosis‐related cell systems and mouse models [127, 128]. These findings support a biologically plausible anti‐endometriotic effect, but they should still be interpreted as preclinical rather than established clinical evidence [129].

Catechins, especially epigallocatechin‐3‐gallate (EGCG) from green tea, are notable for their strong antioxidant and anti‐inflammatory activity. In PMOS‐related animal work, catechins from oolong tea improved ovarian dysfunction, IR, uterine abnormalities, and inflammatory signaling in mice exposed to insulin plus human chorionic gonadotropin, with efficacy compared against metformin in the experimental design [130]. These data support a metabolic and endocrine benefit in PMOS models, although direct clinical evidence in women remains much more limited than the preclinical literature. In endometriosis, the anti‐angiogenic effect of EGCG is much better established experimentally: mouse studies showed that EGCG significantly inhibited lesion development and suppressed vascular signaling, including VEGF/VEGFR (VEGF receptor)‐related pathways, thereby limiting angiogenesis and lesion progression [131, 132]. Reviews consistently describe EGCG as anti‐angiogenic, anti‐fibrotic, antiproliferative, and pro‐apoptotic in endometriosis models, but human trial data are still lacking [133, 134].

Curcumin, the principal polyphenol of turmeric, has similarly broad mechanistic relevance. In PMOS, systematic reviews and meta‐analyses suggest that curcumin can improve glycaemic control and aspects of lipid metabolism, with some randomized controlled trials (RCTs) also reporting favourable effects on body weight, insulin‐related indices, and serum lipids after 12 weeks of supplementation [135, 136, 137]. A 2023 review further summarized experimental and clinical evidence indicating that curcumin may reduce hyperglycaemia, hyperlipidaemia, IR, and hyperandrogenism, although stronger phenotype‐specific trials are still needed [138]. In endometriosis, curcumin has shown anti‐inflammatory, anti‐invasive, anti‐angiogenic, and antiproliferative actions in vitro and in animal studies. Experimental evidence indicates suppression of cytokine signaling, inhibition of invasion and angiogenesis, reduced proliferation of endometrial cells, and induction of cell‐cycle arrest and apoptosis, making curcumin one of the more mechanistically versatile phytochemicals in this field [139, 140]. Thus, resveratrol, quercetin, catechins, and curcumin represent promising plant‐derived antioxidants for both PMOS and endometriosis because they target several shared disease nodes, including OS, inflammation, steroidogenic dysregulation, angiogenesis, and abnormal cellular proliferation. However, the strength of evidence is not uniform across compounds or disorders. Resveratrol and quercetin have some human data in PMOS; catechins and curcumin are supported strongly by mechanistic and animal studies, with curcumin also showing emerging clinical metabolic benefits in PMOS. In endometriosis, most evidence for these compounds remains preclinical, particularly for lesion suppression and anti‐angiogenic action. Therefore, these agents are best described as biologically plausible adjuncts with encouraging translational potential, rather than established stand‐alone therapies, pending larger RCTs with standardized doses, bioavailability‐enhanced formulations, and integrated endocrine‐inflammatory‐redox endpoints [116, 117, 138].

#### 4.1.2 Mechanisms of Action: ROS Scavenging, NF‐κβ Inhibition, Nrf2 Activation, and Antiangiogenesis

As discussed in previous sections, OS and chronic inflammation are central mechanistic nodes in both PMOS and endometriosis. In PMOS, excess ROS, cytokine signaling, mitochondrial dysfunction, and metabolic inflammation contribute to IR, follicular arrest, and steroidogenic dysregulation. In endometriosis, ROS, inflammatory mediators, and aberrant angiogenesis support ectopic lesion survival, invasion, fibrosis, and pain. Against this background, plant‐derived polyphenols such as resveratrol, quercetin, catechins, and curcumin have attracted particular interest because they converge on a limited set of biologically relevant pathways, notably direct ROS scavenging, NF‐κβ suppression, Nrf2 activation, and anti‐angiogenic signaling [12, 117, 128].

The first mechanism is ROS scavenging and reinforcement of endogenous antioxidant defenses. Quercetin and catechins are potent radical scavengers because of their polyhydroxylated structures, and experimental studies indicate that they can reduce LPO while increasing antioxidant enzymes such as SOD, CAT, and GPx. Resveratrol and curcumin likewise help restore redox balance by lowering oxidative damage, preserving mitochondrial function, and improving tissue antioxidant capacity in reproductive and metabolic settings [12, 117, 141]. In PMOS, these redox effects are relevant to follicular development and steroidogenic homeostasis, whereas in endometriosis they may weaken the oxidative microenvironment that favours lesion persistence.

A second major pathway is NF‐κβ inhibition. NF‐κβ is a redox‐sensitive transcription factor that promotes the expression of inflammatory mediators including TNF‐α, IL‐6, IL‐1β, COX‐2, and other molecules involved in cell survival, proliferation, and angiogenesis. This pathway is overactive in both PCOS and endometriosis. Resveratrol, curcumin, and EGCG have each been reported to inhibit NF‐κβ activation by interfering with Iκβ phosphorylation, nuclear translocation, and downstream transcriptional activity; quercetin exerts similar anti‐inflammatory effects in experimental systems. Through this mechanism, these compounds may reduce inflammatory amplification, improve insulin signaling in PMOS, and limit lesion‐supportive inflammation in endometriosis [12, 116, 140, 141].

A third shared mechanism is Nrf2 activation. Nrf2 is a master regulator of cellular antioxidant defense that controls the transcription of detoxifying and cytoprotective enzymes, including HO‐1, NQO1, GSH‐related enzymes, and other redox‐buffering systems. Curcumin, catechins, quercetin, and resveratrol have all been described as natural Nrf2 activators in reproductive or broader oxidative‐stress models, thereby enhancing antioxidant resilience while counterbalancing inflammatory signaling. This mechanism is particularly attractive in PMOS, where oxidative‐metabolic stress is sustained, and in endometriosis, where Nrf2‐linked cytoprotection may blunt local oxidative injury and inflammatory propagation [12, 141, 142].

A fourth and especially relevant mechanism in endometriosis is anti‐angiogenesis. Ectopic endometrial lesions require neovascularization for implantation and growth. Among the compounds discussed, EGCG has some of the strongest experimental anti‐angiogenic evidence, having been shown in mouse models to suppress endometriotic lesion development through inhibition of VEGF‐associated vascular signaling. Resveratrol and curcumin also show anti‐angiogenic activity in endometriosis models, including reduction of VEGF expression, inflammatory angiogenic signaling, and microvessel formation. These vascular effects complement their antioxidant and anti‐inflammatory actions, making them mechanistically attractive for a disease in which inflammation and angiogenesis are tightly coupled [116, 143, 144].

Thus, resveratrol, quercetin, catechins, and curcumin act through overlapping but not identical pathways. Their shared value lies in simultaneously reducing oxidative burden, suppressing NF‐κβ‐driven inflammation, activating Nrf2‐dependent defense programmes, and, particularly in endometriosis, restraining angiogenesis. These mechanisms map closely onto the pathobiology of both PMOS and endometriosis. However, most mechanistic evidence still comes from cell and animal studies, and direct confirmation in well‐powered human trials remains limited. Thus, these compounds are best viewed as mechanistically credible adjuncts with translational promise, rather than fully validated clinical therapies at present [117, 128, 145].

### 4.2 Carotenoids, Vitamins, and Isoflavones

#### 4.2.1 Lycopene, β‐Carotene, Vitamin C, Vitamin E, and Isoflavones

Lycopene is a lipophilic carotenoid that accumulates in lipid‐rich compartments and is an efficient quencher of singlet oxygen and LPO. In endometriosis, a very small exploratory human study reported that lycopene supplementation reduced peritoneal‐fluid MDA, suggesting attenuation of the local oxidative milieu, although the sample size was extremely limited and the finding requires independent confirmation [146]. In PMOS, preclinical evidence is stronger: rat studies have shown that lycopene improves ovarian histopathology, reduces ovarian ROS and ferroptosis‐related injury, and partially restores estrous cyclicity and reproductive hormone balance, supporting a role in lowering oxidative burden relevant to follicular dysfunction [147, 148]. Thus, these data support biological plausibility, but the human evidence remains preliminary, especially for endometriosis.

β‐Carotene and related provitamin‐A carotenoids function both as antioxidants and as precursors of retinoid signaling, thereby linking redox buffering to cellular differentiation and tissue remodeling. Human observational data suggest that carotenoid status may be relevant to endometriosis: an earlier study found altered β‐carotene and lycopene profiles in relation to endometriosis and OS markers, while a more recent population‐based analysis reported that higher serum α‐carotene, β‐carotene, lycopene, and total carotenoids were associated with lower prevalence of endometriosis [149, 150]. However, these are associative rather than interventional data, and direct RCTs of β‐carotene in either endometriosis or PMOS are lacking. Accordingly, causal claims should be avoided; at present, β‐carotene is best discussed as a plausible marker and modulator of oxidative balance rather than a clinically validated treatment.

Vitamin C (ascorbic acid) is a water‐soluble antioxidant that scavenges aqueous radicals and helps regenerate oxidized tocopherol, making it functionally complementary to lipid‐phase antioxidants. In experimental PMOS, vitamin C has shown protective ovarian effects: in a DHEA‐induced rat model, it attenuated ovarian pathophysiology through antioxidant and anti‐apoptotic mechanisms, supporting its relevance to oxidative damage and histologic deterioration in polycystic ovaries [151]. In endometriosis, the clinical literature is more commonly based on combination therapy than monotherapy. A recent meta‐analysis of RCTs concluded that combined vitamin C and vitamin E supplementation improved pain outcomes, including chronic pelvic pain, dysmenorrhaea, and dyspareunia, in women with endometriosis, although the included studies were modest in size and did not always apply harmonized biomarker panels [152]. Thus, vitamin C has mechanistic and adjunctive clinical support, but high‐quality single‐agent RCT evidence in either disorder remains limited.

Vitamin E (α‐tocopherol) is a lipid‐soluble, chain‐breaking antioxidant that protects cell membranes from peroxidative injury. Among the compounds discussed here, vitamin E currently has the strongest interventional signal in PMOS. A 2022 meta‐analysis of 10 randomized trials found that vitamin E, alone or combined with omega‐3 fatty acids or magnesium, significantly lowered triglycerides, low‐density lipoprotein (LDL) cholesterol, total cholesterol, hs‐CRP, and hirsutism score, while increasing NO concentrations; however, the same analysis did not show consistent benefit for all glycaemic or hormonal endpoints [153]. In endometriosis, clinical evidence is smaller but still suggestive: systematic review and meta‐analysis data indicate that vitamin C plus vitamin E can reduce pain symptoms, while earlier individual trials also reported improvement in oxidative and pain‐related outcomes, albeit with heterogeneous designs and modest sample sizes [152, 154]. Overall, vitamin E appears clinically more mature in PMOS than in endometriosis, especially when judged by biomarker‐oriented RCT data.

Isoflavones, particularly soy‐derived compounds such as genistein, are phytoestrogens with selective estrogen‐receptor activity as well as antioxidant and anti‐inflammatory properties. In PMOS, human evidence is available: in a placebo‐controlled trial, 12 weeks of soy isoflavone supplementation improved IR indices, free androgen index, modified Ferriman–Gallwey score, triglycerides, GSH, and MDA, although not all inflammatory or lipid endpoints changed significantly [155]. Earlier clinical observations also suggested reductions in LH, testosterone‐related indices, and some lipid parameters with genistein‐containing interventions, but results across studies have not been uniform [155, 156]. In endometriosis, the evidence is still mainly preclinical. A recent rat study found that soy isoflavones, especially when combined with broccoli extract, reduced implant histopathologic scores and improved OS‐, apoptosis‐, and autophagy‐related gene expression, while a mouse study reported that genistein increased SOD and GPx in peritoneal fluid [157, 158]. These findings support mechanistic promise, but the human endometriosis data remain insufficient for firm therapeutic conclusions.

Overall, the evidence supports strong biologic plausibility for lycopene, β‐carotene, vitamin C, vitamin E, and isoflavones as modulators of OS in PMOS and endometriosis, but the strength of translation differs across compounds. Vitamin E, especially in combination regimens, has the clearest RCT‐based signal in PMOS; soy isoflavones also have human PMOS data with favourable metabolic, androgenic, and oxidative biomarker changes. Lycopene and isoflavones show promising preclinical data in endometriosis, whereas β‐carotene and vitamin C are supported more by mechanistic, observational, or adjunctive‐combination evidence than by large single‐agent trials. Larger, standardized human studies using hard clinical endpoints such as pain, ovulation, fertility, and lesion progression, together with harmonized OS panels, are still needed.

#### 4.2.2 Effects on Systemic OS, Hormonal Modulation, and Inflammatory Markers

Antioxidant nutrients and isoflavones influence three interlinked domains relevant to PMOS and endometriosis: OS, endocrine‐metabolic signaling, and inflammatory tone. Across the literature, the most reproducible effects are biochemical rather than clinical, with clearer improvements in OS markers than in hormonal endpoints; anti‐inflammatory benefits are present but generally modest and often derived from combination regimens rather than single‐agent trials [152, 153, 155].

Systemic and local OS appear to be the most consistently affected domain. In PMOS, meta‐analysis of randomized trials indicates that vitamin E supplementation, either alone or combined with omega‐3 fatty acids or magnesium, reduces oxidative and inflammatory risk markers, including MDA, and improves TAC‐related profiles in several included studies, although not every endpoint changes uniformly across trials [153]. Lycopene shows a more localized oxidative signal: in a very small endometriosis study, lycopene supplementation lowered peritoneal‐fluid MDA, while in PMOS rat models lycopene reduced ovarian ROS, attenuated ovarian pathology, and improved estrous cyclicity and sex‐hormone disturbance, consistent with preferential action in lipid‐rich tissues where carotenoids accumulate [146, 148]. Vitamin C complements lipid‐phase antioxidants by scavenging aqueous radicals and regenerating oxidized tocopherol; in a DHEA‐induced rat model of PMOS, vitamin C reduced ovarian pathophysiology through antioxidant and anti‐apoptotic effects [151]. By contrast, evidence for β‐carotene is mostly observational: altered carotenoid status has been linked to endometriosis‐related oxidative imbalance, and higher serum β‐carotene has been associated with lower endometriosis prevalence in epidemiologic analyses, but direct single‐agent intervention data in PMOS or endometriosis are still lacking [149, 150]. Isoflavones also show redox‐related effects: in women with PMOS, soy isoflavones increased GSH and lowered MDA in a placebo‐controlled trial, and in animal endometriosis models they modulated OS‐related gene expression and antioxidant enzymes [155, 157]. Overall, antioxidant supplementation most reliably improves biochemical OS indices when studies measure reproducible markers such as MDA, GSH, and TAC.

Hormonal modulation is biologically plausible but less consistent. In PMOS, vitamin E supplementation has shown favourable effects on some metabolic and endocrine parameters in pooled analyses, including reductions in hirsutism score and some lipid markers, but attribution is complicated because several trials used combination therapy with omega‐3 fatty acids or magnesium rather than vitamin E alone [153]. Isoflavones have more direct endocrine‐metabolic evidence: in a randomized placebo‐controlled trial, soy isoflavone supplementation for 12 weeks improved IR markers and hormonal status in women with PMOS, including favourable changes in free androgen index and related oxidative biomarkers, although heterogeneity across formulations and study duration remains important [155]. Lycopene restored testosterone, estradiol, LH, and follicle‐stimulating hormone (FSH) in a recent rat PMOS model, but comparable human endocrine data are not yet available [148]. For β‐carotene, endocrine claims should remain cautious because direct randomized evidence in PMOS or endometriosis is essentially absent; current human data are associative rather than causal [149, 150]. Thus, these interventions appear to modify metabolic drivers such as IR more consistently than they normalize the gonadotropin‐androgen axis over short treatment windows.

Inflammatory markers show a modest but credible response. In PMOS, vitamin E‐containing regimens have been associated with lower hs‐CRP and improved inflammatory risk profiles in meta‐analysis, although cytokine‐level data are less consistently reported across trials [153]. In endometriosis, combined antioxidant regimens have shown clinically relevant anti‐inflammatory signals: Santanam et al. reported reductions in chronic pelvic pain together with lower peritoneal‐fluid inflammatory markers, including RANTES (Regulated upon Activation, Normal T‐cell Expressed, and Secreted), IL‐6, and MCP‐1 (monocyte chemoattractant protein‐1), after antioxidant supplementation, while more recent pooled analysis found that vitamin C plus vitamin E reduced dysmenorrhea, dyspareunia, and pelvic pain [152, 159]. Isoflavones also exhibit anti‐inflammatory effects in preclinical endometriosis, with downregulation of lesion‐related OS, autophagy, and apoptosis‐associated gene expression in rat models, but corresponding human biomarker data remain sparse [157]. Vitamin C and carotenoids have anti‐inflammatory activity in experimental settings, but clinical effect sizes are generally smaller and often emerge in the context of combined antioxidant or dietary interventions rather than isolated supplementation [151, 159].

The major limitation of the literature is that most positive signals are biomarker‐based. Improvements in MDA, GSH, TAC, or hs‐CRP do not yet translate consistently into durable patient‐centreed outcomes such as restored fertility, sustained hormonal normalization, lesion regression, or long‐term pain control. Small sample sizes, short follow‐up, co‐supplementation, and heterogeneous doses all complicate inference [152, 153]. Safety at nutritional doses is generally acceptable in the available studies, but higher pharmacologic dosing, especially of fat‐soluble vitamins or concentrated phytoestrogens, still warrants monitoring and better standardization in future trials [153, 155].

Overall, lycopene, vitamin C, vitamin E, and isoflavones show the clearest evidence for reducing OS biomarkers, while inflammatory‐marker improvements are present but usually modest. Hormonal modulation is less uniform and seems to occur more often through improvement of metabolic pathways, particularly IR, than through direct short‐term endocrine resetting. β‐carotene remains the least interventionally validated of the group. Larger, adequately powered randomized trials with standardized dosing, single‐agent arms where feasible, and clinical endpoints alongside harmonized OS and inflammatory panels are needed before these compounds can be translated into firm therapeutic recommendations for PMOS or endometriosis [150, 152, 153].

### 4.3 Herbal Extracts and Traditional Formulations

#### 4.3.1 Cinnamon, Green Tea, Ashwagandha, Spearmint, and Ginseng

Several botanicals have emerged as adjunctive candidates for PMOS and, to a lesser extent, endometriosis because they combine antioxidant, anti‐inflammatory, metabolic, and endocrine‐modulating properties. However, the strength of evidence is uneven across agents. For PMOS, the most credible human data currently support cinnamon, spearmint, and green tea for selected metabolic or androgen‐related endpoints, whereas for endometriosis the evidence is largely preclinical for green tea and ginseng, and remains very limited for cinnamon, ashwagandha, and spearmint [133, 160, 161].

Cinnamon (*Cinnamomum* spp.) has the strongest clinical rationale in PMOS among the botanicals listed here because of its insulin‐sensitizing effects. In women with PMOS, randomized trials have shown that cinnamon supplementation can improve insulin sensitivity, reduce fasting insulin, and may improve menstrual cyclicity, although the magnitude of benefit varies across studies and sample sizes are modest [162, 163, 164]. Mechanistically, these effects are thought to relate to improved insulin signaling and downstream metabolic control rather than direct ovarian endocrine resetting. At present, clinically relevant evidence for cinnamon in endometriosis is sparse, so its role should be framed primarily within PMOS rather than generalized across both disorders [162, 163].

Green tea (*Camellia sinensis*), particularly through EGCG, has antioxidant, anti‐inflammatory, and anti‐angiogenic potential. In PMOS, clinical and meta‐analytic evidence suggests that green tea or tea‐derived supplements may reduce body weight and improve fasting insulin, fasting glucose, and, in some studies, free testosterone, although hormonal effects are less consistent than metabolic ones [56, 160, 165]. In endometriosis, the evidence is stronger mechanistically than clinically: EGCG inhibits angiogenesis and suppresses VEGFC/VEGFR2 signaling in experimental endometriosis, and reviews support anti‐fibrotic, pro‐apoptotic, and lesion‐suppressive effects in preclinical models [131, 133, 166]. The statement that green tea may improve an estrogen‐dominant environment via aromatase modulation is biologically plausible, but the more solid endometriosis evidence currently centrrs on anti‐angiogenic and antiproliferative actions rather than direct clinical aromatase‐driven benefit [131, 166].

Ashwagandha (*Withania somnifera*) is often discussed as a stress‐modulating and antioxidant botanical, but direct evidence in PMOS remains limited and is mostly preclinical. In a letrozole‐induced rat model, a combination of *W. somnifera* and *Tribulus terrestris* normalized estrous cyclicity, increased FSH, reduced elevated LH, estradiol, and testosterone, lowered cholesterol, and improved ovarian histology [167]. These findings are promising, but they do not establish the efficacy of ashwagandha alone, because the intervention was a botanical combination rather than isolated *W. somnifera*. Human clinical evidence specific to PMOS remains insufficient. There is currently no meaningful direct evidence for ashwagandha as a treatment for endometriosis. Therefore, this herb should be presented as mechanistically interesting but clinically preliminary in reproductive disorders [167].

Spearmint (*Mentha spicata*) is best supported as an anti‐androgenic adjunct in PMOS. In an RCT, spearmint herbal tea significantly reduced free and total testosterone over 30 days and improved self‐reported hirsutism‐related quality of life measures, although objective Ferriman–Gallwey scores did not significantly change during the short study period [168]. This pattern suggests a genuine endocrine signal but also underscores that short trials may be too brief to capture changes in terminal hair growth. Direct evidence for spearmint in endometriosis is lacking. Any suggestion that it may alleviate endometriosis‐related pain should therefore be framed as speculative and based on general anti‐inflammatory potential, not disease‐specific trial data [168].

Ginseng (*Panax ginseng* and related species) has antioxidants and anti‐inflammatory properties mediated largely by ginsenosides. In PMOS models, Korean red ginseng has improved ovarian morphology, reduced cyst formation, lowered LH, and alleviated oxidative‐inflammatory changes in estradiol valerate‐ and DHEA‐induced rat models, supporting a plausible role in ovarian functional recovery [169, 170, 171]. In endometriosis, the evidence is largely preclinical and review‐based: red ginseng and specific ginsenoside‐centred studies suggest anti‐inflammatory, antiproliferative, and immunomodulatory effects, including regulation of endometriosis‐related miRNA expression and inflammatory pathways [62, 161, 172]. However, robust human clinical trials in either PMOS or endometriosis are still lacking, so ginseng should be considered promising but not clinically established [161, 169].

Thus, cinnamon, green tea, spearmint, ashwagandha, and ginseng are best viewed as adjunctive, mechanism‐rich botanicals rather than validated stand‐alone therapies for PMOS or endometriosis. Cinnamon has the clearest evidence for insulin‐related improvement in PMOS; spearmint has the most specific anti‐androgenic human signal; green tea has supportive metabolic data in PMOS and strong anti‐angiogenic preclinical data in endometriosis; ginseng is supported mainly by animal and mechanistic studies; and ashwagandha remains the least clinically validated in this context. Thus, while these herbs are often described as multi‐targeted and generally well tolerated in short‐term studies, claims of broad efficacy across both disorders should remain cautious until larger, better‐standardized randomized trials are available [133, 160, 161].

#### 4.3.2 Evidence From Animal Models and Clinical Studies in PMOS and Endometriosis

Building on the mechanistic profiles outlined above, the translational strength of these botanicals varies considerably across conditions and study types. Overall, the evidence is more mature in PMOS than in endometriosis, with a clearer progression from preclinical models to human studies.

Cinnamon shows one of the most consistent translational patterns in PMOS, with both animal and clinical studies indicating improvements in metabolic parameters and, to a lesser extent, reproductive function. These findings support its role as an adjunct particularly in IR associated phenotypes, although evidence for direct endocrine effects remains limited [162, 163, 164, 173].

In contrast, ashwagandha remains at an early stage of investigation. As discussed above, available preclinical data are derived from combination interventions rather than isolated compounds, and clinical evidence is currently insufficient. Its role should therefore be considered preliminary and hypothesis generating rather than evidence based [167].

Spearmint demonstrates a more specific endocrine signal in PMOS, particularly in relation to androgen modulation. While short term clinical findings support biochemical effects, the lack of longer duration studies limits conclusions regarding sustained clinical outcomes. Evidence in endometriosis remains absent [168].

Ginseng represents a mechanistically promising but predominantly preclinical candidate across both disorders. Experimental studies support anti‐inflammatory, anti‐proliferative, and hormone modulating effects, including impacts on lesion biology in endometriosis and ovarian function in PMOS. However, the absence of robust human trials limits its current clinical applicability [169, 170, 171, 172, 174, 175, 176]. Thus, these data indicate that while several botanicals demonstrate biologically plausible and experimentally supported effects, clinical evidence remains uneven. PMOS currently has the strongest translational support, particularly for cinnamon and spearmint, whereas in endometriosis the evidence is largely confined to preclinical models. Future studies should prioritize well designed clinical trials that distinguish metabolic, endocrine, inflammatory, and patient centred outcomes to better define the therapeutic role of these agents.

### 4.4 Nanoformulations and Bioavailability Enhancement Strategies

The therapeutic utility of plant‐derived antioxidants in PMOS and endometriosis is frequently limited by poor aqueous solubility, chemical instability, rapid intestinal and hepatic metabolism, and low tissue exposure after oral administration. These pharmacokinetic constraints are especially relevant for polyphenols such as curcumin, resveratrol, quercetin, and EGCG, whose *in vitro* antioxidant and anti‐inflammatory activities often exceed what can be reproduced *in vivo* with conventional formulations [23, 177, 178]. Accordingly, nanotechnology‐based delivery systems, including liposomes, polymeric or lipid nanoparticles, and phytosome‐type phospholipid complexes, have emerged as rational strategies to improve stability, absorption, circulation time, and, potentially, reproductive‐tissue exposure [23, 178, 179].

Among these, liposomal formulations are attractive because phospholipid vesicles can encapsulate both hydrophilic and lipophilic compounds, shield them from degradation, and facilitate membrane interaction. In the broader polyphenol literature, liposomes consistently improve the pharmacokinetic behaviour of poorly bioavailable phytochemicals, including curcumin, resveratrol, and quercetin [180, 181]. However, for PMOS and endometriosis specifically, direct evidence is still limited. Current reviews support the concept that liposomal delivery could enhance reproductive applications, but most disease‐specific data remain preclinical or extrapolated from gynaecologic and oncology models rather than from large dedicated PMOS or endometriosis studies [177, 182]. Therefore, it is more accurate to state that liposomes are a promising platform for improving antioxidant delivery in these disorders, rather than claiming that liposomal curcumin, resveratrol, or quercetin has already demonstrated clear clinical efficacy in PMOS or endometriosis [181, 182].

Nanoparticle‐based carriers provide an additional level of formulation control. Polymeric nanoparticles such as PLGA [Poly(lactic‐co‐glycolic acid)] systems, solid lipid nanoparticles, and related nanocarriers can improve solubility, prolong exposure, and sometimes enhance local cellular uptake. In endometriosis, a frequently cited preclinical strategy combined letrozole and curcumin in PLGA nanoparticles, with reported improvement in OS parameters, matrix‐remodeling markers, and angiogenesis‐related features in experimental disease models; similarly, broader reviews of endometriosis nanomedicine describe multiple nanoparticle approaches that improve lesion targeting and bioactivity in preclinical settings [182, 183]. In PMOS, recent reviews emphasize that nanoencapsulation is being explored to improve ovarian accumulation and efficacy of polyphenols such as curcumin and quercetin, but much of this work remains at the proof‐of‐concept or animal‐study stage rather than at the level of mature translational evidence [177, 184]. Thus, the statement that “curcumin‐loaded PLGA nanoparticles restore mitochondrial integrity in ovarian tissue” is directionally plausible, but should be framed cautiously unless tied to a specific primary study; the more defensible general claim is that nanoparticle formulations may enhance antioxidant and anti‐inflammatory effects relative to free compounds in preclinical PMOS models [177, 184].

Phytosomes represent a somewhat different strategy, in which phytochemicals are complexed with phospholipids to improve membrane permeability and intestinal absorption without major chemical modification of the parent molecule. Reviews consistently describe phytosomes as effective bioavailability enhancers for poorly absorbed polyphenols, including curcumin, quercetin, and resveratrol, with improved systemic exposure reported across multiple non‐gynecologic contexts [178, 179, 185]. However, direct phytosome‐focused evidence in endometriosis and PMOS remains scarce. For this reason, phytosomes should be presented as a physiologically compatible and translationally attractive delivery approach, but not yet as a validated reproductive therapeutic platform [178, 185]. The same caution applies to “hybrid” nanocarriers: they are conceptually promising, but evidence in PMOS or endometriosis is still emerging rather than established.

A balanced interpretation is therefore warranted. The core problem of poor bioavailability is well established for curcumin, resveratrol, quercetin, and related plant antioxidants, and nanocarrier systems do appear capable of improving stability, absorption, and functional delivery [181, 186, 187]. What remains underdeveloped is the disease‐specific translational bridge: there are still too few standardized studies examining pharmacokinetics, biodistribution to ovarian or endometriotic tissue, dose‐response relationships, long‐term safety, and comparative efficacy against conventional formulations in women with PMOS or endometriosis [177, 182]. Concerns around manufacturing reproducibility, carrier toxicity, biodegradability, and regulatory standardization also remain relevant, especially for metallic or more complex multifunctional nanosystems [182, 188].

Future work should therefore prioritize standardized formulation protocols, full pharmacokinetic characterization, reproductive‐tissue biodistribution studies, and phenotype‐specific efficacy testing in PMOS and endometriosis. If these gaps are addressed, nanocarrier‐assisted delivery could move plant‐derived antioxidants beyond the level of dietary adjuncts and toward clinically actionable therapeutics with improved redox, inflammatory, and endocrine targeting [177, 181, 184]. At present, though, the evidence supports strong pharmaceutical rationale and encourageing preclinical potential, rather than definitive clinical translation.

### 4.5 Comparative Efficacy and Safety Considerations

Plant‐derived antioxidants such as curcumin, resveratrol, quercetin, EGCG, and lycopene are mechanistically attractive in PMOS and endometriosis because they converge on OS, inflammatory signaling, mitochondrial dysfunction, and, in some cases, angiogenesis or steroidogenic regulation. However, their comparative efficacy is difficult to rank with confidence, because literature is heterogeneous in formulation, dose, treatment duration, disease phenotype, and endpoint selection. At present, most favourable signals are biomarker‐based or preclinical rather than based on robust head‐to‐head clinical comparisons. In general, curcumin, resveratrol, quercetin, and EGCG show strong mechanistic activity but are limited by poor oral bioavailability, whereas lycopene appears more compartment‐specific and has a narrower direct intervention base in reproductive disorders. Thus, the question is less whether these compounds are biologically active, and more whether adequate exposure can be achieved safely and reproducibly in the relevant metabolic or reproductive‐tissue context [141, 181, 189].

A major determinant of benefit is dose‐dependence. Many polyphenols exhibit hormetic behaviour, with antioxidant and cytoprotective effects at lower concentrations but pro‐oxidant or cytotoxic effects at higher concentrations. This pattern has been described particularly for curcumin and resveratrol, whose redox effects can shift according to concentration, cell type, and oxidative background. Curcumin is widely described as protective at lower micromolar exposure but capable of promoting oxidative injury, cell‐cycle arrest, or apoptosis at higher concentrations; similarly, resveratrol can behave as an antioxidant at modest exposure yet acquire prooxidant features at higher doses or under specific metal‐rich or highly reactive cellular conditions [190, 191, 192]. This is highly relevant to PMOS and endometriosis, because both are chronic disorders in which long‐term supplementation may be contemplated, and dose‐escalation does not necessarily translate into greater therapeutic benefit.

Hormonal context also matters, especially for compounds with phytoestrogenic or receptor‐modulating properties. Resveratrol is particularly important here: it has been described as a phytoestrogen with dose‐dependent and context‐dependent estrogen receptor effects, and reviews in endometriosis note that low‐dose versus high‐dose exposure may not have equivalent endocrine consequences. In estrogen‐dependent disorders such as endometriosis, this means that the same compound may theoretically confer anti‐inflammatory or anti‐angiogenic benefit while still requiring careful attention to hormonal milieu, baseline estrogen signaling, and concomitant therapy. The available clinical evidence does not support a blanket statement that resveratrol is uniformly beneficial across all endometriosis phenotypes; indeed, at least one placebo‐controlled study found no superiority over placebo for pain when added to oral contraceptive therapy [116, 193, 194].

Potential drug‐phytochemical interactions also deserve attention. Reviews note that catechins, curcumin, resveratrol, quercetin, and related compounds can inhibit or modify CYP‐mediated metabolism, although the magnitude and clinical relevance vary by compound and exposure level. This is relevant to women using oral contraceptives, metformin, analgesics, or anti‐inflammatory drugs in PMOS or endometriosis management, because co‐administration could theoretically alter drug exposure or tolerability. The interaction literature is stronger as a pharmacologic caution than as proof of common clinically severe events in these specific disorders, so the prudent interpretation is that combined use should be monitored rather than automatically avoided [190, 195, 196].

For EGCG, safety considerations are somewhat more concrete. EGCG has recognized antioxidant and anti‐inflammatory potential, but its oral bioavailability is limited, and high‐dose supplemental use has been associated in broader clinical literature with hepatotoxicity risk in susceptible individuals. In addition, green tea catechins can impair non‐heme iron absorption, a point that may be clinically relevant in menstruating women or in patients already vulnerable to iron deficiency. These concerns do not negate the therapeutic value of EGCG, but they do argue against assuming that “natural” equates to risk‐free, especially when concentrated extracts are used over longer periods or alongside other medications [197, 198, 199].

Short‐term trials often report acceptable tolerability and favourable changes in oxidative or metabolic biomarkers, but chronic exposure raises unresolved questions about gastrointestinal intolerance, micronutrient interactions, cumulative hepatic burden, and interindividual variation in metabolism and tissue exposure. This uncertainty is compounded by bioavailability‐enhancement strategies, such as nanoformulations or co‐formulation with absorption enhancers, which may improve efficacy but could also shift safety thresholds relative to conventional dietary intake [178, 181, 189]. Thus, plant‐derived antioxidants remain promising adjuncts rather than interchangeable therapies. Their safest and most scientifically defensible use lies in precision‐guided application: selecting compounds according to disease phenotype, endocrine context, likely interaction burden, formulation quality, and achievable tissue exposure. Future studies should move beyond broad supplementation paradigms and define therapeutic windows, comparative efficacy, interaction profiles, and long‐term safety in phenotype‐stratified PMOS and endometriosis cohorts before these agents can be recommended with high clinical confidence [181, 190, 194].

## 5 Integrative Perspective: Microbial and Plant‐Derived Antioxidants as Bioactive Cocktails

### 5.1 Rationale for Combined Probiotic–Phytochemical Interventions

Combining probiotics with plant‐derived phytochemicals may provide a systems‐level strategy to modulate redox‐inflammatory signaling in PMOS and endometriosis. This rationale is grounded in the gut‐reproductive axis, in which microbial metabolites, endocrine signaling, mucosal barrier integrity, and immune mediators interact bidirectionally with reproductive tissues. In both disorders, gut dysbiosis, impaired barrier function, altered SCFA signaling, and chronic low‐grade inflammation have been implicated in IR, estrogen dysregulation, and OS, although the strength of causal evidence remains greater for PMOS than for endometriosis [66, 200, 201] (Figure 1).

Probiotic strains, especially Lactobacillus and Bifidobacterium, may partially restore microbial equilibrium, and dampen inflammatory signaling through SCFA‐related mechanisms, including activation of GPCRs and histone deacetylase inhibition. In PMOS, randomized trials and systematic reviews indicate that probiotics, prebiotics, and synbiotics can improve IR indices and selected inflammatory or oxidative biomarkers, although strain‐specific responses and study heterogeneity remain important limitations [95, 202]. In endometriosis, probiotics remain clinically less developed, but mechanistic and early human studies suggest possible benefit through immune regulation, pain reduction, and modulation of inflammatory tone [75, 99, 101].

Phytochemicals such as curcumin, resveratrol, quercetin, and catechins interact reciprocally with the gut microbiota. On one hand, polyphenols can enrich beneficial taxa, suppress potentially pathogenic organisms, and favour metabolite profiles associated with lower oxidative and inflammatory burden. On the other hand, gut microbes biotransform polyphenols into smaller metabolites that may have greater bioavailability or altered biological potency than the parent compounds [203, 204, 205]. Curcumin and resveratrol, in particular, have been repeatedly discussed as compounds whose biological effects depend partly on microbial metabolism and microbiota remodeling [206, 207, 208].

From this perspective, a probiotic‐phytochemical combination is biologically plausible because each component may reinforce the other. Probiotics may improve intestinal metabolism and barrier conditions that favour polyphenol stability, transformation, and downstream signaling, whereas phytochemicals may promote a microbiota configuration more compatible with SCFA production, endotoxin control, and mucosal redox balance. However, this should be stated carefully: direct clinical evidence for probiotic‐polyphenol co‐supplementation in PMOS or endometriosis is still sparse. Most available data come from parallel rather than integrated intervention literatures, or from in vitro fermentation and non‐gynecologic models showing that mixtures of probiotic strains with quercetin, resveratrol, or other polyphenols can beneficially alter microbial composition and metabolic output [203, 204, 209]. These interactions provide a mechanistic rationale for combined interventions, while their effects in PMOS and endometriosis require direct clinical evaluation.

In PMOS, this combined framework is attractive because both probiotics and polyphenols independently improve aspects of insulin sensitivity, OS, and inflammatory tone. Systematic reviews suggest that microbiota‐targeted supplementation can improve HOMA‐IR and related metabolic endpoints, while meta‐analyses of polyphenols report reductions in serum insulin, body mass index (BMI), and, in some cases, LH or androgen‐related variables [95, 210]. It is therefore reasonable to hypothesize that co‐modulation of gut microbes and polyphenol metabolism could produce additive or synergistic effects on IR, adipokine dysregulation, and redox imbalance. Yet this remains a hypothesis requiring direct testing rather than a clinically established conclusion.

In endometriosis, combined interventions could be investigated for their effects on pain and lesion‐associated processes. Probiotics may attenuate immune dysregulation and pain, while phytochemicals such as curcumin, EGCG, quercetin, and resveratrol target NF‐κB signaling, angiogenesis, oxidative injury, and lesion proliferation in preclinical systems [75, 133, 144]. Because endometriosis appears linked to gut dysbiosis, endotoxin signaling, and altered estrogen‐related microbial metabolism, co‐supplementation offers a testable approach to targeting microbial and lesion‐associated pathways concurrently. However, controlled trials specifically evaluating probiotic–phytochemical combinations in endometriosis are not yet available, so any statement of therapeutic synergy must remain inferential rather than evidence‐based.

A further practical opportunity is that probiotics may help overcome one of the major limitations of polyphenol therapy: poor and variable bioavailability. Since many phytochemicals are extensively metabolized by the gut microbiota, modifying the intestinal ecosystem may influence their absorption, transformation, and systemic persistence. Even so, the notion that probiotics “prevent intestinal degradation” of polyphenols is too simplistic; in reality, microbial metabolism may either enhance or reduce bioactivity depending on the compound, host microbiota, and metabolite formed [203, 205, 208]. Therefore, future work should focus less on generic co‐supplementation and more on identifying strain‐compound compatibility, metabolite signatures, and host phenotypes that predict benefit.

The central translational question is whether a defined combination improves a clinically relevant outcome beyond the effects of its individual components. The combined nutraceutical study in healthy and hypertensive participants provides evidence outside the reproductive disease setting, whereas the PMOS polyphenol meta‐analysis evaluates phytochemical interventions rather than probiotic–phytochemical combinations (Brasil et al., 2025; Balieiro et al., 2024). A randomized factorial trial comparing placebo, the bacterial preparation, the phytochemical, and their combination could estimate component effects and test a prespecified interaction. Synergy would require evidence of an interaction on the specified outcome scale; superiority of the combination over either component alone would not by itself establish it.

### 5.2 Phenotype‐Guided Trial Design

The concept of personalized nutrition offers a plausible framework for optimizing probiotic and phytochemical interventions in both PMOS and endometriosis, because neither disorder is biologically uniform. In PMOS, trial stratification should characterize IR, adiposity, androgen status, and ovulatory function directly, because body size alone does not define metabolic or reproductive phenotype [211]. These characteristics can inform eligibility, baseline adjustment, and prespecified subgroup analyses [212]. In endometriosis, lesion subtype, hormonal treatment, pain characteristics, and fertility goals provide corresponding clinical dimensions for evaluating response [213].

Molecular profiling can complement this clinical characterization. Microbiome composition and metabolite measurements may identify functional differences relevant to phytochemical metabolism or microbial intervention responses [203], while redox measures can characterize biochemical effects. Genetic associations with disease susceptibility provide candidate variables for investigation, but treatment selection requires evidence that a marker predicts differential benefit from a defined intervention. Microbial and genetic predictors should therefore be evaluated prospectively before being used to guide supplementation [212].

A practical trial framework begins with a defined disease population, a standardized preparation, and a prespecified clinical outcome. Baseline phenotypes can then be used to test response variation, with microbial, metabolic, and redox measurements serving as secondary mechanistic outcomes. Candidate predictors identified through exploratory analyses require independent validation [212]. This sequence connects biological heterogeneity with reproducible treatment selection and provides a foundation for personalized adjunctive care.

Genetic variation in antioxidant and inflammatory pathways offers a further dimension for investigating biological heterogeneity. PMOS susceptibility research has examined genes involved in redox regulation, including *SOD2*, *GPX1*, *GPX4*, and *CAT* [214, 215], alongside cytokine‐gene variants involving TNF‐α and interleukins [212, 216]. In endometriosis, studies have investigated associations between OS‐related variants, including *GPX1*, *CAT*, *GSTM1*, and *GSTT1*, and infertility‐associated disease [217]. These findings identify candidate pathways for mechanistic investigation; their relevance to intervention response requires separate evaluation. Genomic measures could be incorporated as exploratory variables in probiotic and phytochemical trials, with treatment–genotype interactions prespecified where supported by prior evidence and candidate predictors independently validated [212]. Increased endometrial *NQO1* expression in women with PMOS represents a distinct molecular observation and should be evaluated separately from inherited variation [218].

### 5.3 Translational Challenges and Future Research Priorities

Despite growing experimental and early clinical interest in probiotics and phytochemicals as redox‐modulating adjuncts in PMOS and endometriosis, translation into routine care remains limited by the quality and structure of the evidence base. Most intervention studies are small, short‐term, and methodologically heterogeneous, with substantial variation in strains, formulations, doses, co‐supplementation, baseline phenotype, and outcome selection. In PMOS, recent systematic reviews of probiotics, prebiotics, and synbiotics conclude that while metabolic and inflammatory improvements are plausible, heterogeneity across trials still prevents firm clinical standardization. In endometriosis, the clinical evidence is even less mature, with only a small number of probiotic trials and a much larger reliance on mechanistic or animal data. This is reflected at the guideline level: the 2023 International Evidence‐based Guideline for PCOS does not position probiotics or phytochemicals as core evidence‐based therapy, and the 2022 ESHRE endometriosis guideline notes that good‐quality proof for complementary approaches remains limited [75, 95, 219, 220, 221].

A second major barrier is the lack of formulation and dose standardization. Probiotic efficacy is strain‐specific and depends on viability, colonization potential, ecological compatibility with the host microbiome, and manufacturing quality. Phytochemicals face a parallel problem: extraction method, molecular form, food matrix, and co‐administered compounds can all markedly alter bioavailability and biological effect. This is especially relevant for curcumin, resveratrol, quercetin, and EGCG, whose pharmacologic promise is often greater than their conventional oral exposure. Reviews on nanocarriers, phytosomes, and other bioavailability‐enhancing systems suggest that encapsulation and advanced delivery may improve translational performance, but these technologies themselves require pharmacokinetic, toxicologic, and reproducibility validation before broad clinical adoption [177, 178, 181, 182].

Another challenge is biological heterogeneity, which likely underlies much of the inconsistency across current trials. PMOS includes metabolically distinct phenotypes, such as lean and obese forms, that differ in IR burden, adiposity, inflammatory tone, and androgen excess. Endometriosis also spans biologically different entities, including superficial peritoneal disease, endometrioma, and deep infiltrating disease, with different inflammatory, fibrotic, and pain‐related signatures. It is therefore unlikely that a single probiotic strain, synbiotic combination, or phytochemical regimen will perform uniformly across all patients. Future studies need phenotype‐stratified designs and should incorporate unified redox, inflammatory, endocrine, and metabolic readouts rather than relying mainly on isolated biomarkers or symptom scores. Integrating metagenomics, metabolomics, and, where feasible, nutrigenomic profiling could help identify responder subgroups and define strain‐compound‐host compatibility more precisely [201, 219, 222, 223].

The final translational hurdle is clinical integration and regulation. To become part of evidence‐guided reproductive care, probiotics and phytochemicals will need more than mechanistic plausibility; they will require multicentre RCTs with standardized formulations, dose‐ranging arms, longer follow‐up, safety monitoring, and clinically meaningful endpoints such as ovulation, fertility, pain reduction, lesion behaviour, recurrence, and quality of life. They will also need clearer regulatory definitions, especially for live biotherapeutics, synbiotics, and bioavailability‐enhanced phytochemical formulations. Until then, these agents are best regarded as promising adjuncts with real biologic rationale, but not yet as established components of mainstream PMOS or endometriosis management. The next step in the field is not simply to accumulate more small trials, but to build precision, phenotype‐aware translational pipelines that can move microbial and phytochemical interventions from supportive supplementation toward reproducible therapeutic application [95, 177, 219, 220].

## 6 Conclusions

OS and inflammation connect endocrine, metabolic, and immune disturbances with reproductive dysfunction in PMOS and endometriosis. Their contributions differ by disease context: IR and androgen excess predominate in PMOS, whereas estrogen‐dependent lesion biology and local inflammatory remodeling characterize endometriosis. Mitochondrial dysfunction, cytokine signaling, and altered antioxidant responses provide overlapping downstream targets for investigating microbial and plant‐derived interventions.

Selected probiotic preparations and phytochemicals have produced improvements in metabolic, endocrine, oxidative–inflammatory, or pain‐related outcomes, with evidence varying across interventions and disorders. Experimental findings involving microbial metabolites, NF‐κB signaling, and Nrf2‐regulated responses help explain potential mechanisms, while their contribution to clinical benefit requires direct evaluation. Sustained effects on ovulation, fertility, lesion progression, and recurrence remain insufficiently established, and complementary mechanisms alone do not demonstrate therapeutic synergy.

Clinical translation should connect standardized preparations and dose selection with prespecified disease‐specific outcomes, adequate follow‐up, and safety assessment. Trials incorporating metabolic and reproductive phenotypes alongside microbial and redox measurements can determine which patients benefit and whether biochemical responses accompany meaningful clinical improvement. This approach provides a foundation for establishing the role of microbial and plant‐derived interventions in adjunctive reproductive care.

## Author Contributions

**Afnan Athar Hussain:** writing – original draft, methodology, data curation. **Shafna Mohamed Ali:** methodology, writing – original draft, data curation. **Ruaa Safwan Al Naser:** writing – original draft, methodology, data curation. **Sahar Safari Abdullah:** writing – original draft, methodology, data curation. **Mohamad Imadeddin Barakat:** writing – original draft, methodology, data curation. **Sulagna Dutta:** conceptualization, writing – review and editing. **Pallav Sengupta:** conceptualization, writing – review and editing, supervision. **Motamarri Venkata Naga Lalitha Chaitanya:** writing – review and editing. **Prashanth Hegde:** writing – review and editing. **Shatabhisha Chakrabarti:** writing – review and editing. **Israel Maldonado Rosas:** writing – review and editing. **Shubhadeep Roychoudhury:** conceptualization, writing – review and editing, supervision.

## Ethics Statement

The authors have nothing to report.

## Conflicts of Interest

The authors declare no conflicts of interest.

## Data Availability

The authors have nothing to report.
